# The Social Aspects of Illness

**Published:** 1904-06-25

**Authors:** 


### The Hospital.

A Journal of

The Medical Sciences and Hospital Administration.

Vol. XXXVI.—No. 926. JUNE 25, 1904.

### The Social Aspects of Illness.

Among many interesting papers read at the Conference on Sick Children to which we lately called attention, one eminently deserving of special notice was contributed by Dr. Newsholme, the medical officer of health for Brighton, on the subject of "The Social Aspects of the Prevention of Illness." The object of the author was to call attention to the difficulties arising from the fact that the duty of preserving health, more especially in the case of children, while its effective discharge is a matter of supreme importance to the community, is yet primarily a duty of individuals, which cannot be undertaken by the community without a dangerous weakening of the sense of individual responsibility. A half-starved child, by reason of its proneness to fall a victim to some infectious disease, and to convey it to a school, may easily become the centre and source of a dangerous epidemic; and so far the community, for its own protection, may justifiably insist that all children in attendance at school shall be adequately fed, and shall be protected, as far as may be possible, against current contagions. Such insistence, however, may be of such a kind as to carry with it the practical release of parents from one of the most imperative of their duties, the duty of taking proper care of the children they have been the instruments of bringing into the world. If the community, for its own protection, sees fit to undertake the performance of duties which parents of the lowest class now habitually neglect, those parents will be the first to recognise that a burden which they ought to bear has been removed from their shoulders, and to acquiesce cordially in its removal. If, on the other hand, the community suffers the upspringing and constant reinforcement of bands of half-starved and otherwise neglected children, it is providing for the maintenance and increase of a sickly, and eventually of a criminal, population, and is thereby permitting, if not actively encouraging, the growth of evils which will ultimately recoil upon itself. A great deal which passes muster for charity is open to the objection that all it does is to apply feeble or partial antidotes to fully-developed mischiefs which should have been grappled with in their origin; and this objection may be said to apply, at least to some extent, even to charities which in their general operations may be regarded as entirely beneficent. Every hospital is to some extent an assurance to the working classes that they may dispense with any endeavour to make provision against sickness; and every meal given to half-starved school children is an assurance to self-indulgent parents that they may safely spend upon drink and tobacco the money which ought to provide food and clothing for their families. Is there any means by which the necessities of the case can be supplied, while at the same time neglectful parents are made to suffer for their neglect?

Dr. Newsholme quotes Messrs. Rowntree, Sherwell, and Whittaker in support of the assertion that, excluding abstainers, each working-class family spends on an average about 7s. a week in alcoholic drinks; and points out that, even if no family were to exceed this average amount, an enormous fund would be available for the better nourishment of children if the money thus employed could be diverted to right uses. He makes no mention of expenditure on tobacco, which, as wives and children do not as a rule at all participate in its results, while they sometimes do get a drink of beer, must be regarded as an entirely selfish outlay on the part of the father. Resting the case on the consumption of beer and gin alone, it seems to show, beyond dispute, that many working-class parents fail to recognise that the duties which they owe to their children are duties which they owe also to the State, and for the neglect of which the State may rightly punish them. Society and the law, so far, have done little or nothing to bring home this elementary truth to those whom it most concerns, except in cases of cruelty or neglect of such a kind as to bring life into immediate peril; and Dr. Newsholme advocates that the principle applicable to these cases should be extended to those of a less serious character, and should be made to cover all instances in which it could be shown that parents were not making proper provision for their children according to their means. He suggests as a test that all the children attending free schools should be periodically weighed under the supervision of the teachers; and that the failure of a child to increase in weight in due proportion to its years should be held to constitute a case for inquiry. In the first instance, a medical inspection should be made; and, if this did not disclose any physical cause for the failure, or if collateral circumstances known to the teacher or the school-attendance officer indicated the probability of neglect, a domestic inspection should follow. Dr. Newsholme admits that this might be "somewhat inquisitorial" in character, as it might, and often would, involve investigation into the total wages of the family and into the proportion of them spent at home; but he does not regard this as a serious objection. It would bring home to careless, lazy, and dissolute parents their responsibility before the law; while the school weighings would afford a reason for timely and therefore effective intervention. Dr. Newsholme anticipates that legal proceedings would seldom be required, inasmuch as warning notices pointing out the condition of the children and indicating the remedy would alone exert an immense influence for good. In cases where cruel and continued neglect was proved, he thinks it would be within the limits of human ingenuity to extend the provisions of a recent enactment, and to devise some means of so identifying the offending parents that no publican would dare to supply them with strong drink. We believe the operations of the "black list," so far, have not been eminently successful, but the suggestion, if it could be carried out in practice, would probably be exceedingly effective. Dr. Newsholme is undoubtedly right in declaring that "compulsory abstinence is the real remedy"; but, even beyond this, the fear of being marked in the manner indicated would certainly exert a powerful influence. The proposed inquiries, moreover, would have the incidental effect of distinguishing the cases calling for charity from those calling for punishment, and would leave the benevolent at full liberty to deal with the former, while removing all risk of giving encouragement to the latter.

